# ARIA-Italy multidisciplinary consensus on nasal polyposis and biological treatments: Update 2025^☆^^☆☆^

**DOI:** 10.1016/j.waojou.2025.101058

**Published:** 2025-05-09

**Authors:** Carlo Lombardi, Rikki F. Canevari, Diego Bagnasco, Maria Beatrice Bilò, Giorgio Walter Canonica, Cristiano Caruso, Paolo Castelnuovo, Lorenzo Cecchi, Orietta Calcinoni, Mauro Carone, Marco Cassatella, Renato Cutrera, Gianni Danesi, Eugenio De Corso, Mario Di Gioacchino, Fabiano Di Marco, Stefania Gallo, Matteo Gelardi, Enrico Heffler, Clara Imperatore, Cristiana Indolfi, Massimo Landi, Amelia Licari, Francesco Liotta, Alberto Macchi, Luca Malvezzi, Sara Manti, Francesco Menzella, Claudio Micheletto, Fabio Midulla, Michele Miraglia Del Giudice, Antonino Musarra, Vincenzo Patella, Diego Peroni, Laura Petrarca, Giorgio Piacentini, Alessandro Pusateri, Veronica Seccia, Gianenrico Senna, Adriano Vaghi, Carlo Vancheri, Giovanni Passalacqua, ARIA Italy, ARIA-ITALY PANEL, S. Arasi, D. Bagnasco, I. Baiardini, S. Barbaglia, F. Barosso, R. Bernardini, A. Berti, B. Bilò, L. Brussino, F.R. Canevari, G.W. Canonica, C. Caruso, A.A. Caruso, A.G. Caviglia, L. Cecchi, P. Comberiati, L. Cosmi, M. Cottini, M. Crivellaro, D. Di Bona, A. Fiocchi, S. Frateiacci, M. Gelardi, G. Guida, E. Heffler, C. Incorvaia, M. Landi, M.A. Licari, L. Malvezzi, M. Milanese, F. Mina, E. Nettis, G. Pala, V. Patella, D. Peroni, S. Peveri, F. Puggioni, A. Pusateri, O. Rossi, E. Ridolo, E. Savi, J. Schroeder, V. Seccia, G.E. Senna, M. Triggiani, M. Uberti, G. Varricchi, M.T. Ventura, M.T. Zedda

**Affiliations:** a Unit of Allergology, Clinical Immunology & Respiratory Diseases, Fondazione Poliambulanza Istituto Ospedaliero, Brescia, Italy; b Dipartimento DISC, Clinica Otorinolaringoiatria, IRCCS Policlinico San Martino, Università di Genova, Italy; c Allergy and Respiratory Diseases, IRCCS PoliclinicoS.Martino, University of Genoa, Italy; d Dipartimento di Scienze Cliniche e Molecolari (DISCLIMO), Università Politecnica delle Marche, Azienda Ospedaliero-Universitaria delle Marche, Ancona, Italy; e Department of Biomedical Science-Humanitas University-Pieve Emanuele (MI), Personalized Medicine, Asthma and Allergy-IRCCS Humanitas Research Hospital-Rozzano (NI), Italy; f UOSD DH Internal Medicine and Digestive Diseases, Fondazione Policlinico A. Gemelli, IRCCS, Università Cattolica del Sacro Cuore, Rome Italy; g DBSV, Clinica ORL, Università Insubria, ASST-Settelaghi, Varese, Italy; h SOS Allergologia e Immunologia Clinica Prato, USL Toscana Centro, Italy; i Società Italiana Liberi Professionisti, Centro Medico Turati, Milan, Italy; j Istituti Clinici Scientifici Maugeri, IRCCS Bari, UOC di Pneumologia, Bari, Italy; k Società Italiana di Immunologia Clinica e Allergologia.PatologIa Generale, University of Italy, Italy; l Pediatric Pulmonology and Cystic Fibrosis Unit Respiratory Intermediate Care Unit, Sleep and Long Term Ventilation Unit, ERN - Lung Reference Center for Rare Respiratory diseases Bambino Gesù Children's Hospital, IRCCS, Rome, Italy; m UnitàOperativa di Otorinolaringoiatria e Microchirurgia della Base Cranica, Ospedale Papa Giovanni XXIII, Bergamo, Italy; n Otorhinolaryngology Unit, A. Gemelli University Hospital IRCCS, Rome, Italy; o Società Italiana di Asma Allergologia e Immunologia Clinica, University of Chieti, Italy; p Dipartimento di Scienze della Salute, Università degli Studi di Milano, ASST Papa Giovanni XXIII, Bergamo, Italy; q Ospedale di Circolo e Fondazione Macchi, Varese, Italy; r ORL Clinic, University of Foggia, Italy; s AICNA, Asl Napoli 1 Centro Distretto 24 L. Crispi, Italy; t Dipartimento della Donna, del Bambino e di Chirurgia Generale e Specialistica, Università degli Studi della Campania “Luigi Vanvitelli” Italy; u Department of Medical Sciences - Graduate School of Allergology and Clinical Immunology University of Turin, Italy; v SC Pediatric Clinic, Fondazione IRCCS Policlinico San Matteo, University of Pavia, Pavia, Italy; w Dipartimento di Medicina Sperimentale e Clinica, Università di Firenze, Italy; x Otorinolaringoiatria e Chirurgia Cervico Facciale – Humanitas San Pio X Ambulatorio II Livello Rino-Allergologico IRCCS HumanitasResearch Hospital, Italy; y Medico Chirurgo, Pediatra, Dottore di Ricerca, Ricercatore Tempo Determinato UOC Pediatria, AOU G. Martino, Università di Messina, Italy; z UOC Pneumologia Distretto di Asolo, AULSS2 Marca Trevigiana, Italy; aa UOC di Pneumologia, Azienda Ospedaliera Universitaria Verona, Veneto, Italy; ab Dipartimento Materno Infantile e Scienze Urologiche Policlinico Umberto I, Roma, Italy; ac Servizio di Allergologia, Casa della Salute di Scilla, ASP di Reggio Calabria, Italy; ad UOC di Medicina Interna Ospedale Civile “Santa Maria della Speranza”, Salerno, Italy; ae Department of Clinical and Experimental Medicine, University of Pisa, Italy; af Department of Maternal Infantile and Urological Science, Università“La Sapienza, Roma, Italy; ag UOC di Pediatria, AOUI Verona, Dipartimento di Scienze Chirurgiche, Odontostomatologiche e Materno-Infantili, Università di Verona, Italy; ah UOC Otorinolaringoiatria ASST Papa Giovanni XXIII Bergamo, Italy; ai ASP 6, Palermo, Italy; aj Department of Medicine, Asthma Center, University of Verona, Italy; ak ASST RodhensePneumologia, Italy; al UOC Malattiedell’ApparatoRespiratorio, Università degli Studi di Catania, Italy

**Keywords:** Chronic rhinosinusitis with nasal polyposis, Severe asthma, Biological agents, Monoclonal antibodies, ARIA (allergic rhinitis and its impact on asthma), Personalized medicine

## Abstract

In recent years, it was recognized that type-2 inflammation connects nasal polyposis and severe asthma (SA) in addition to other type-2 diseases. Thus, some biological drugs developed for SA appeared to exert a favourable effect also in nasal polyposis. So far, there are several trials supporting this concept; therefore, some monoclonal antibodies already used for SA were assessed also in chronic rhinosinusistis with nasal polyposis (CRSwNP), with promising results.

Since different specialists are involved in the management of nasal polyposis (eg, pulmonologists, ENT specialists, allergists, immunologists, pediatricians), it was felt that an updated educational and informative document was needed to better identify the indications of biological therapies in nasal polyposis. We collected the main Italian scientific societies, and prepared (under the umbrella of Allergic Rhinitis and its Impact on Asthma, ARIA) a document endorsed by all societies, to provide a provisional statement for the future use of monoclonal antibodies (MAbs) as a medical treatment for polyposis, possibly associated with SA. The above mentioned document was the first endorsed document on this aspect, and the additional evidence required an update. The current pathogenic knowledge and the experimental evidence are herein reviewed, and some suggestions for a correct prescription and follow-up are provided.

## Introduction and historical context

Chronic rhinosinusitis with nasal polyposis (CRSwNP) is a worldwide, highly prevalent disease which may have important healthcare implications and relevant social costs.**^1^** Despite the fact that CRSwNP is usually a relatively easy-to-diagnose disease, several aspects of this pathology remain in a grey zone, expecially after the introduction of monoclonal antibodies (MAbs), such as the aetiology, the association with different asthma phenotypes, and epidemiology. In addition, until today, the treatment became a more challenging aspect for specialists: up to few years ago, the available therapeutic options for CRSwNP were essentially topical or oral systemic corticosteroids (OCS) and surgery. Recently, due to the more detailed knowledge of the responsible CRSwNP pathogenic mechanisms, several MAbs have become available for the treatment of severe asthma (SA). These MAbs, acting essentially on Type-2 inflammation, were tested also in CNRwNP. When it was recognized that also this disease frequently shared the same pathomechanisms of SA (type 2 inflammation), the biological therapies were tested also in CRSwNP, and some of the MAbs initially studied for the treatment of SA were approved by regulatory authorities also for nasal polyposis. The research on this topic is currently ongoing. Specific randomized clinical trials, or real-life trials were performed and published, with the aim of assessing the effect of omalizumab (anti-IgE), benralizumab (anti IL-5 receptor), mepolizumab (anti circulating IL-5), reslizumab (anti circulating IL-5), tezepelumab (anti thymic stromal lymphopoietin (TSLP), and dupilumab (anti IL-4 receptor α) in CRSwNP, with promising results. Due to the overlapping clinical nature of CRSwNP, different specialists are involved in the clinical management: allergists, pulmonologists, otolaryngologists, clinical immunologists, and pediatricians. Therefore, it is important to ensure that all of them have a full knowledge of the pathogenic mechanisms and the new therapeutic perspectives, promoting a common approach to the use and prescription of MAbs therapy in CRwNP. In this context, the Italian panel of Allergic Rhinitis and its Impact on Asthma (ARIA) experts invited the different scientific societies involved in this field to participate in the preparation of an informative and educational document on the use of MAbs in CRSwNP. The first version was published in 2021,**^2^** and the present one represents its update. The full panel of ARIA-Italy, which approved the document, is available at http://www.progetto-aria.it. Each Society, on behalf of their presidents, identified experts who contributed equally to the article and approved the final version Table I.

**Table 1:** “Participant Societies and identified experts”

SOCIETY	PRESIDENT	DEFINITION	NOMINATED EXPERTS
AAITO	Lorenzo Cecchi lorenzo.cecchi@unifi.it	Associazione Allergologi Italiani Territoriali e Ospedalieri	Maria Beatrice Bilò Antonino Musarra
AIOLP	Orietta Calcinoni orietta.calcinoni@gmail.com	Associazione Italiana Otorinolaringoiatri Libero- Professionisti	None provided
AICNA	Pietro Pecoraro segreteria@aicna.it	AccademiaItaliana di CitologiaNasale	Enrico Heffler, Clara Imperatore, Veronica Seccia Matteo Gelardi
AIPO	Mauro Carone mauro.carone@icsmaugeri.it aoiposegreteria@aiporicerche.it	AssociazioneItalianaPneumologiOspedalieri	Claudio Micheletto Adriano Vaghi
ARIA CHAIRMAN	Carlo Lombardi lombardicarlo@libero.it	Allergic Rhinitis and its Impact on Asthma– ITALY	Giorgio Walter Canonica Vincenzo Patella Diego Bagnasco
ARIA CHAIRMAN	Giovanni Passalacqua passalacqua@unige.it	Allergic Rhinitis and its Impact on Asthma - ITALY	Massimo Landi Giorgio Piacentini Diego Peroni
IAR	Rikki F.Canevari canevari@edu.unige.it	Italian Academy of Rhinology	Eugenio De Corso Stefania Gallo Paolo Castelnuovo
SIAAIC	Mario Di Gioacchino digioacchino@me.com	Società Italiana Asma Allergia e Immunologia Clinica	Cristiano Caruso Gianenrico Senna
SIAIP	Michele Miraglia Dal Giudice michele.miraglia@unicampania.it	Società Italiana Allergologia Immunologia Pediatrica	Amelia Licari Cristiana Indolfi
SIICA	Marco Cassatella marco.cassatella@univr.it	Società Italiana Immunologia Clinica e Allergologia	Francesco Liotta
SIMRI	Fabio Midulla midulla@uniroma1.it	Società Italiana Malattie Respiratorie Infantili	Sara Manti Laura Petrarca
SioeChCF	Gianni Danesi gianni.danesi@me.com	SocietàItalianadi otorinolaringologia e chirurgiacervico-facciale (SioeChCF)	Alessandro Pusateri
SIP	Annamaria Staiano staiano@unina.it presidenza@sip.it	Società Italiana Pediatria	Renato Cutrera Fabio Midulla
SIP/IRS	Fabiano di Marco fabiano.dimarco@unimi.it	Società Italiana Pneumologia	Carlo Vancheri Francesco Menzella

## Framework and extent of the problem

Chronic rhinosinusitis is an inflammatory disease of the nose and paranasal sinuses, which may be present with nasal polyposis (CRSwNP) or without (CRSsNP). Nasal polyposis is a part of the CRSwNP framework, as indicated by the European Position Paper on Rhinosinusitis and Nasal Polyps (EPOS). Hence, the terms nasal polyposis or CRSwNP can be considered substantially equivalent.^3^ However, when referring to nasal polyposis, it is important to keep in mind that it is part of a wider spectrum of diseases, where inflammation is frequently the pivotal part of the problem. Data from epidemiological studies show that the prevalence of CRSwNP varies from 2% to 14%, depending on the geographical area and the diagnostic criteria^4^, ^5^, ^6^, ^7^. Concerning CRSwNP, the estimated prevalence is approximately 1–5%, again with a variability based on geographical areas.^8^ Randomized controlled trials and real-life data suggest that asthma is present in 30–60% of individuals with CRSwNP, while CRSwNP is present in up to 70% of patients with bronchial asthma.^9^, ^10^, ^11^ The overlap and influence of various factors and comorbidities, including asthma, aspirin sensitivity, atopic dermatitis, allergy, and cigarette smoking, make a precise classification of nasal polyposis still difficult to be made. In addition, also other diseases, such as fungal rhinosinusitis, some forms of vasculitis, and cystic fibrosis are sometimes associated with polyposis. The role of allergy in polyposis was the subject of a long-standing debate. Until a century ago, allergy (allergic rhinitis, in particular) was thought to be a direct cause of nasal polyposis, but more recent studies showed that polyposis is present in the same proportion of allergic individuals (approximately 2–4%) as it is in the general population.^12^^,^^13^ In contrast, if there is a high prevalence of atopic individuals with polyposis there is not, so far, enough evidence to conclude that atopy plays a causal role in the pathogenesis.^14^ At present, the only “strong” correlations observed were those between rhinitis and asthma and between severe asthma (SA) and CRSwNP,^11^ but not between CRSwNP and allergic rhinitis.

CRSwNP reduces the quality of life of those affected, and the symptom of “nasal obstruction” certainly remains the major one responsible for discomfort felt in everyday life, and is associated with hyposmia/anosmia and sleep disturbances. We should also not forget the costs related to the need of repeated surgery, and those due to the side effects of the use of nasal and/or oral corticosteroids (OCS), often taken for long periods: osteoporosis, diabetes, cataracts, obesity, hypertension, glaucoma, and delayed growth (in childhood).^15^, ^16^, ^17^

## Clinical aspects and current therapy

The main symptoms/signs variably associated with CRSwNP are: nasal obstruction, anterior/posterior nasal discharge (even purulent), facial pain (frontal, maxillary), hypo/anosmia, and sleep disturbances. Dysgeusia and a perception of ear muffling or cough can also be present. As mentioned above, asthma, often severe, is present in a significant proportion of patients, and hypersensitivity to aspirin or non-steroidal anti-inflammatory drugs (NSAIDs) can be detected in about one-third of patients. The characteristic clinical presentation and evolution of the disease is that of a progressive worsening overtime. Hyposmia/anosmia often occurs early and could be a sign of massive polyposis or of severe inflammation. By definition, 2 or more symptoms, lasting at least 12 weeks, must be present: one of them must be nasal obstruction or rhinorrhea (anterior or posterior), added to facial pain or hyposmia. In addition to the above-mentioned clinical aspects, objective findings are mandatory for diagnosis and follow-up. Nasal endoscopy and/or computed tomography (CT) scan of the paranasal sinuses is required, while conventional x-ray of the facial region is neither useful nor diagnostic. In this regard, there are staging systems to quantify the severity of the disease. The most commonly used and accepted are the Nasal Polyp Score (NPS) (endoscopic) and the Lund-McKay (CT-scan) scale (Table II A-B).^18^^,^^19^ The aforementioned are commonly used in clinical regulatory trials, in addition to subjective tests to explore for instance the olfactory perception.^20^

**Table 2:** “ Objective evaluation for nasal polyposis”

A Nasal Polyp Score (NPS) a
0 = no polyps
1 = small polyps in middle meatus/edema
2 = middle meatus blocked
3 = polyps extending beyond the middle meatus without complete obstruction, or extending to the sphenoethmoidal recess
4 = massive nasal polyposis

B Lund-Mackay CT staging (each side separately) b

Maxillary (0, 1, 2)
Anterior ethmoid (0, 1, 2)
Posterior ethmoid (0, 1, 2)
Sphenoid (0, 1, 2)
Frontal (0, 1, 2)
Osteomeatal complex (0, 2)

a The NPS scale ranges from 0 (no polyp) to 4 (large polyps) for each nostril. These scores are tallied from each nostril for a total score ranging from 0 to 8.

b Each side is graded separately. A combined score of up to 24 ispossible. Of note, an aplastic (absent) frontal sinus receives a score of 0

In the latest clinical trials, exploring the efficacy of biologicals in CRSwNP, the disease was usually defined as NPS ≥5 (with a score of ≥2 for each nasal cavity). The parameter defined and validated in the literature and most frequently used to determine the impact of symptoms on the quality of life (QoL) of patients and establish whether the disease is uncontrolled is the Sino-Nasal Outcome Test (SNOT-22).^21^ This is a questionnaire self-completed by the patient: the maximum score is 110 (greatest disease impact) and the minimum clinically important difference is 8.9 points. A score >50 usually indicates severe polyposis. Other methods of evaluation can be used in association with the main criteria to quantify the severity of polyposis and its perceived impact such as: Visual Analog Scale (VAS), a continuous graphic scale of severity from 0 to 10; the UPSIT/Sniffing test that evaluates the sense of smell by recognising or not of standard aromas; and the peak nasal inspiratory flow that easily evaluates the nasal patency. Another specialistic parameter is the so-called ASSESS score that evaluates the efficacy and extension of the previous surgical interventions. ^22^

The current usual treatment of CRSwNP is based on nasal irrigation with saline or nasal steroids, OCS, and endoscopic surgery (ESS). In principle, nasal steroids are used to reduce the growth of polyps, to delay surgery, or to prevent relapse after surgery.^23^ In treatment cycles or as continuous treatment, OCS are used in the event of massive, relapsing, or disabling polyposis. OCS usually are effective to treat symptoms and hyposmia but, due to their known side effects, a continuous use is not recommended,^17^^,^^24^ as well it is proscribed the use of depot steroid preparations. Despite the well-known long-term side effects of this kind of therapeutic approach, repeated use of OCS in clinical practice remains common, used far more frequently and for much longer periods than recommended by the EPOS guidelines (2 weeks)^23^^,^^24^ and reviews.^17^

Nevertheless, ESS remains the standard treatment to improve patency of paranasal sinus ostia, followed by treatment with a nasal steroid. The aim of surgical approach is limited to unblocking the nasal cavities and opening the ostia of the paranasal sinuses, to restore ventilation and allow intranasal corticosteroids to reach the mucous membrane of the sinuses. The underlying problem remains the high incidence of relapse after surgery which, in turn, would require the use of systemic steroids. Regarding children under 10 years of age, CRSwNP is rare, and their presence should prompt an assessment, at first instance, the presence of congenital diseases (ie, cystic fibrosis and ciliary dyskinesia). The cornerstone of CRS therapy in children is medical treatment with appropriate antibiotic therapy and treatment of comorbidities such as allergic rhinitis and asthma. Surgery is justified only in a small percentage of children. Antibiotic therapy is the same as that for acute rhinosinusitis, but for a longer duration, typically 3–4 weeks. The choice of antimicrobial should include active agents against *Staphylococcus aureus*. At present, the efficacy and safety of MAbs as a therapy for CRSwNP has been largely confirmed.^25^ Understanding the immunological basis of CRSwNP provides a highly effective alternative for patients with type 2 inflammation and a refractory, uncontrolled course through the use of MAbs.

## Pathophysiology and immunology

In recent years, our understanding of the pathophysiological and immunological mechanisms underlying NP rapidly evolved, and “type 2” inflammation is now recognized as the common denominator of some forms of CRSwNP and also asthma. Type 2 inflammation is driven by both parts of the immune system: the innate immune system (innate lymphoid cells 2, ILC2) and the acquired immune system (Th2 cells). Studies carried out on severe asthma have identified specific characteristics linked to the T2-prevalent “component” of the disease: the involvement of ILC2 and of cytokines called alarmins (TSLP, IL-25, IL-23), as early effectors in response to the possible epithelial damage. The complex immunological machine is then activated, with T helper 2 lymphocytes, innate lymphoid cells 2, and their typical cytokines (IL-4, IL-5, and IL-13).

In particular:^26^^,^^27^

• Specific IgE are the trigger of the allergic reaction and activate mast cell degranulation following contact with the allergen;
• IL-4 regulates the differentiation, traffic and migration of naive T cells into Th2 cells;
• IL-5 promotes the maturation, activation, and survival of eosinophils;
• IL-13 is involved in hyperplasia of goblet cells, mucus production, and mucociliary differentiation of nasal epithelial cells;
• IL-4 and IL-13 both play a role in the class switching of B cells to IgE production; they stimulate eosinophil trafficking to the tissues, induce synthesis of chemokines (eotaxine-3,TARC) and expression of adhesion molecules that promote migration of inflammatory cells tothe site of inflammation;
• TSLP, as an alarmin, reacts to epithelial damage and starts the drive towards T2 inflammation via ILC2 and Th2 lymphocytes.

**Fig. 1:**
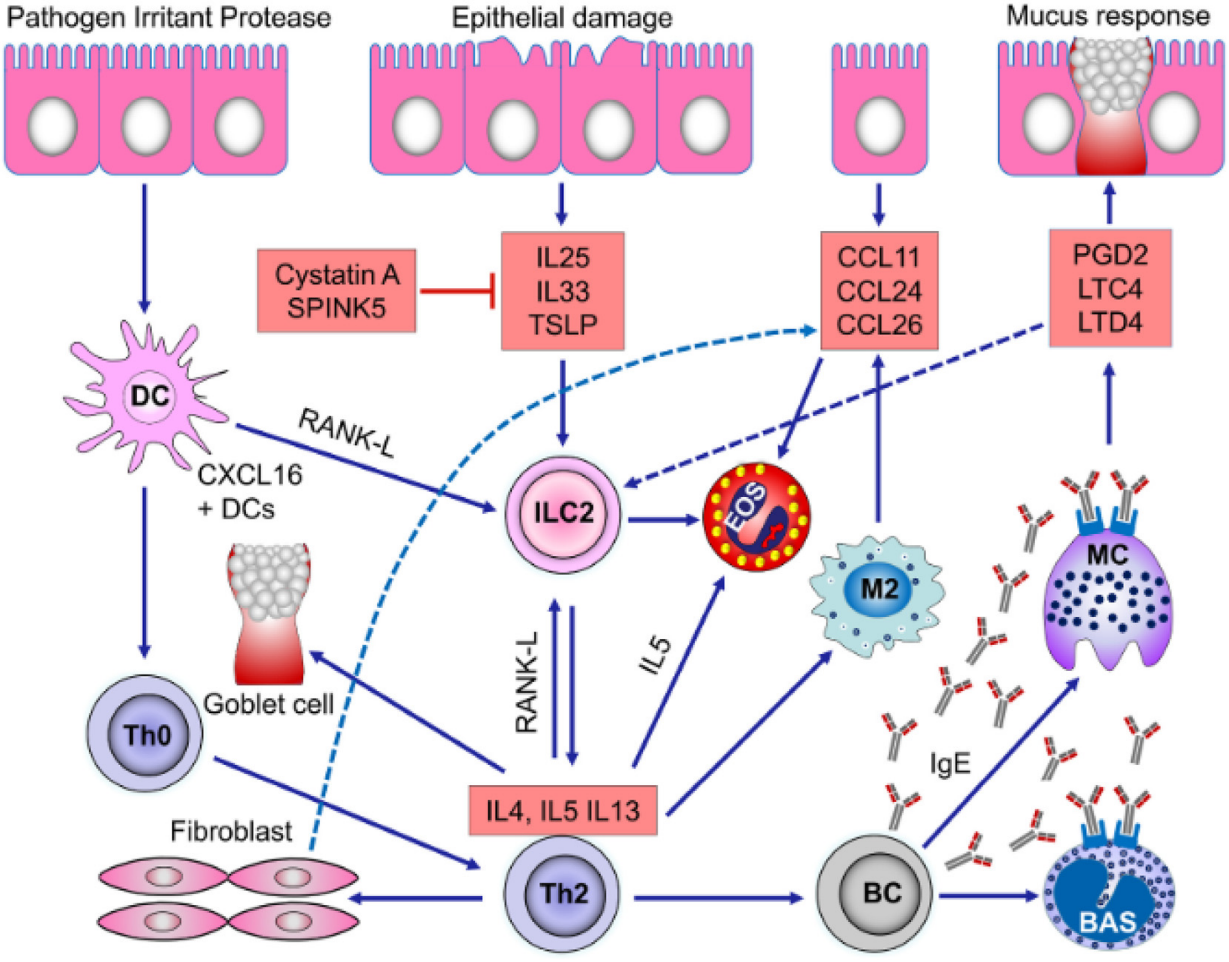
“CRSwNP pathogenetic mechanisms” *from Shah SA et al.*^26^

In this context, allergy, superantigens, and aspirin intolerance mechanisms can be also involved.^28^, ^29^, ^30^ The described inflammation mechanisms, despite there is now a “grey zone”, are common inflammatory paths (namely, type 2) shared by some forms of asthma and nasal polyposis (Fig. 1). A distinctive feature is the presence and activation of eosinophils (which predominantly infiltrate the polyps in this type of inflammation). This characteristic is not observed, for instance, in antrochoanal polyps and polyps associated with cystic fibrosis or ciliary dyskinesia, where a TH1/TH17 polarization with neutrophilic inflammation tends to prevail.^31^ Type 2 inflammation, common to both asthma and polyposis, is anyway apparent.

## CRSwNP and biomarkers

The identification of specific biomarkers is crucial for understanding the CRSwNP pathogenesis, stratifying patients, and optimizing clinical management.^32^ The most studied biomarkers for endotyping CRS are eosinophils, immunoglobulin E (IgE), periostin, and nasal nitric oxide (nNO). These biomarkers are obtained from peripheral blood, nasal secretions, tissue biopsies from the paranasal sinuses, and nasal exhaled breath sample, respectively.^33^ Blood eosinophils have shown significant utility as biomarkers for the diagnosis, prognosis, and monitoring of CRSwNP, but their use is limited by a lack of specificity, as elevated levels can occur in other inflammatory conditions.^34^ Although blood eosinophilia is a minimally invasive biomarker, it does not always correlate with tissue eosinophilia. Despite these considerations, both blood and tissue eosinophil levels play a crucial role for prognosis. More precisely a blood count higher than 0.455 × 10^9^/L is associated with a greater risk of needing prolonged systemic therapy after surgery.^35^ Tissue eosinophilia is also associated with greater disease severity and an elevated risk of recurrence. Eosinophil levels exceeding 55 and 70 eosinophils per high-power field (HPF) represent an useful cutoff for predicting recurrence within 2 years post-surgery.^36^^,^^37^ Furthermore, the eosinophilic CRSwNP endotype respond better to corticosteroids than non-eosinophilic ones.^38^^,^^39^ The role of serum total and specific IgE is debated. The EPOS and European Forum for Research and Education in Allergy and Airway Diseases (EUFOREA) guidelines^24^^,^^26^ recommend the measurement of total IgE and eosinophils for patient endotyping. However, a recent study has demonstrated a higher predictive value for clinical outcomes when using specific IgEs compared to total IgEs, and the detection of IgEs in nasal polyps have could be a potential marker of eosinophilic inflammation, as they since directly promote eosinophil migration at the tissue level.^40^ Furthermore, a recent study revealed that the anti-IgE biologic agent omalizumab reduces tissue IgE, leading to a decrease in inflammation, hence tissue IgE could serve as a predictive marker for the response to omalizumab.^41^ IL-5 in CRS is also a prognostic and predictive biomarker. It has been shown that IL-5 expression was markedly increased in the sinonasal regions in patients with eosinophilic chronic rhinosinusitis.^42^ From a prognostic perspective, IL-5 was found to be elevated in patients with CRSwNP who had a higher risk of recurrence.^43^ Turner et al investigated the levels of TH2 cytokines (namely IL-4/5/13) in nasal secretions and found that elevated IL-5 and IL-13 were not only found in CRS when compared to healthy controls, they were associated with worse objective measures of disease severity and greater rates of revision surgery.^34^^,^^44^

IL-25, IL-33 and TSLP are also epithelial-derived cytokine that are important in the pathogenesis of CRSwNP and asthma, and may be potential relevant biomarkers for CRSwNP.^45^^,^^46^

Current studies have shown that eosinophilic cationic protein (ECP) could serve as a biomarker for the assessment and monitoring of patients with eosinophilic CRS. A recent study demonstrated that ECP levels are associated with an increased risk of postoperative recurrence in patients with endotype T2-CRS.^47^

Periostin is a protein involved in tissue remodeling, produced by epithelial cells in response to IL-4 and IL-13. Recent works suggest that periostin could be useful both for its prognostic value in identifying disease severity and its predictive value for surgical treatment.^34^ Exhaled oral nitric oxide (FeNO) is currently used as a marker of asthma control – with lower levels correlating with better disease control. Nasal nitric oxide (nNO) has been shown to be useful as a potential noninvasive prognostic and predictive biomarker in patients with CRSwNP. nNO is decreased in patients with nasal polyposis, and studies have attributed this phenomenon to nasal polyps resulting in sinus ostial occlusion, preventing NO within the sinus cavities from being excreted via the nasal cavities into the external environment.^48^^,^^49^ Studies have also suggested a possible role of nNO to be an objective means of quantifying the severity of CRSwNP non-invasively. Other biomarkers, such matrix metalloproteinases, P-glycoprotein, glucocorticoid receptor, bitter and sweet taste tests, and various T-cell markers may have a role. Finally, Cope et al demonstrated specific microbiota classifications that correlated with patient phenotypes, including the presence of nasal polyps.^50^ A higher than expected proportion of patients with CRSwNP are colonized with *Staphylococcus aureus*, and IgE antibodies to *S. aureus* enterotoxins are frequently found in diseased tissue specimens.^51^

## MAbs in nasal polyposis: Clinical trials

As previously mentioned, asthma is a disease that is frequently sustained by a type 2 inflammatory cascade, and it represented the study prototype in precision medicine for identifying and characterizing the best treatments. In this model, the cytokines IL-4, IL-5, IL-13, and the alarmin, thymic stromal lymphopoietin (TSLP), are essentially involved as well as IgE. Because many forms of NP also share this same type of inflammation, some of the biologic drugs currently available for SA (anti-IgE, anti-IL4Ra, anti-IL5, anti-IL5Ra, and anti TSLP) were approved also in CRwNP by the European Medicines Agency (EMA), US Food and Drug Administration (FDA), and in many other countries.^52^, ^53^, ^54^, ^55^, ^56^, ^57^, ^58^, ^59^, ^60^ In the case of nasal polyposis, the experimental data were firstly based on case reports (off-label use), or small trials, but these were followed by robust randomized double-blind placebo control trials, specifically designed forCRSwNP. A summary of the registration trials is reported in Table 3.^52^, ^53^, ^54^, ^55^, ^56^, ^57^, ^58^, ^59^, ^60^ As apparent, not all trials evaluated the same primary goal nor used the same time frame, but the clinical results were overall comparable.

**Table 3:** Main registration trials for MAbs in NP (↓ Significant improvement)

AUTHOR (ref)	DRUG	DOSE	DURAT	ACT/PLA	MAIN RESULTS
Gevaert ^33^	Omalizumab	150–375 mg/mth	16 wks	16/8	↓ in total NPS and Lund-Mackay CT score
Gevaert ^34^	Omalizumab	76–500 mg every 2–4 wks	24 wks	134/132	↓ in NPS, SNOT-22 score
Gevaert ^35^	Mepolizumab	750 mg i,v, 2 inj 28 days apart	8 wks	20/10	↓ in NPS
Bachert ^36^	Mepolizumab	750 mg i.v. every 4 wks	25 wks	54/51	↓ in surgery need ↓ in NPS
Bachert ^37^	Mepolizumab	100 mg s.q./month	52wks	206/201	↓ in NPS
Gevaert ^38^	Reslizumab	1–3 mg/kg i,v	12 wks	16/8	Treatment with 1 mg/kg improved NPS up to 12 weeks in 5 of 8 subjects. In the 3 mg/kg treatment group 4 of 8 pts had a better NPS nasal up to 4 weeks after treatment; however, there was a deterioration of NPS in 4 pts 12 weeks after receiving reslizumab.
Bachert ^39^	Dupilumab	600 mg load + 300 mg/wk	16 wks	30/30	↓ in NPS ↓ Lund-Mackay CT S ↓ SinoNasal Outcome Test ↓ in UPSIT
Bachert ^40^	Dupilumab	300 mg s.q. every 2 wks	24 wks	143/133	↓ in total NPS and Lund-Mackay CT score
Bachert ^40^	Dupilumab	300 mg s,q, every 2 wks then 300 mg s.q. every 4 wks	52 wks	150-153/153	↓ in total NPS and Lund-Mackay CT score
Laidlaw ^41^ (asthma ± NP)	Tezepelumab	210 mg/4wks	56 wks	525/526	↓ SNOT-22
Lipworth ^83^	Tezepelumab	210 mg/4 wks	52 wks	203/205	↓ in NPS ↓ Lund-Mackay CT S ↓ SNOT-22 ↓ Nasal-congestion- Score ↓ loss-smell-score ↓ in UPSIT Significant reduction in OCS use in teze group.

## Real-world and post-hoc analyses

Several declared post-hoc analyses or partial analyses from the regulatory trials, in addition to long term evaluations and meta-analyses were performed. In these cases, the comparisons were never done head-to-head, by using the same endpoints, so that a direct comparison against biologicals was always indirect. For instance there was no difference among biologicals, because timeframe, primary endpoints, and dosages were different. 61, 62, 63 These meta-analyses show that the 3 biological agents approved for the treatment of the CRSwNP (dupilumab, mepolizumab, and omalizumab) are superior to placebo about many outcomes and parameters, as nasal obstruction, loss of smell, NPS, need for ESS, use of systemic corticosteroids, and QOL. A very recent network meta-analysis conducted by Wang et al compared a comprehensive set of 19 studies evaluating the efficacy of 4 distinct biologics treatments.^64^ This reticulated meta-analysis confirmed that: (a) dupilumab, omalizumab, benralizumab, and mepolizumab were superior to placebo from the nasal polyp score(NPS); (b) dupilumab was superior to placebo from the SNOT-22 score; and (c) dupilumab and omalizumab were superior to placebo from the nasal congestion severity (NCS). Finally, in terms of cumulative sorting under the surface of the curve (SUCRA) values, dupilumab was the best performer in the NPS (0.92), SNOT-22 score (0.70), and NCS (0.93).

Even more important remain the follow-up studies, where the placebo groups switched to active treatment, or when discontinuation of the active was attempted.^65^, ^66^, ^67^, ^68^, ^69^, ^70^ All these studies (open label follow-up or real-life observations confirmed the long-term efficacy of MAbs, but did not answer the question on when to discontinue the treatment. Certainly those studies demonstrated that the biological treatments could reduce significantly the intake of OCS.

## The need for an interdisciplinary network

It is now well clear that Type 2 inflammation is shared by various diseases such as severe asthma, CRSwNP, eosinophilic granulomatosis with poliangytis, eosinophilic esophagitis, eosinophilic pneumonia and hypereosinophilic syndrome. Therefore, more and more strict interdisciplinary cooperation is needed, to share the different diagnostic approaches and therapeutic strategies.^71^

We report herein, as an example, what we obtained from the Ligurian Healthcare Agency (ALISA), to establish an order in the prescription of biologicals for CRSwNP, and this was the first accepted initiative in Italy,^72^ (Table 4). We hope that initiative could be expanded, in order to get an optimal cooperation between the specialists who have to manage SA, CRSwNP and other T2 diseases.

**Table 4:** [ref. 72] “Evaluation flow-chart for the prescription of biologicals for CRSwNP (ALISA ligurian deliberation 160)”

	BASELINE	1st admin.	1 MO	3 MO	6 MO	12 MO
Nasal endoscopy with NASAL POLYP SCORE	X		X	X	X	X
Facial CT with Lund MacKay SCORE end ACCESS SCORE	X					X
Number/type of previous surgery	X					
Olphactometry 16 & VAS	X		X	X	X	X
SNOT 22	X		X	X	X	X
Nasal steroids in the last year (and ongoing treatment)	X		X	X	X	X
Systemic steroid in the last year (and ongoing treatment)	X		X	X	X	X
Evaluation of possible adverse events from systemic steroids	X					X
Asthma Control Test (ACT)	X	X	X	X	X	X
Spirometry and pulmonologic evaluation (if asthma symptoms)	X					X
Allergy evaluation (Skin Prick Test, specific IgE)	X					
Peripheraleosinophilia	X		X	X	X	X
Total IgE	X					X
Blood cortisol (if > 2 courses systemic steroid in the last year or continous)	X					X
Adverseevents monitoring		X	X	X	X	X
Fraction Exhaled Nitric Oxide (FeNO). On decision	X					X
Nasal cytology. On decision	X					X

## Proposals, and what remains to be defined

**Fig. 2:**
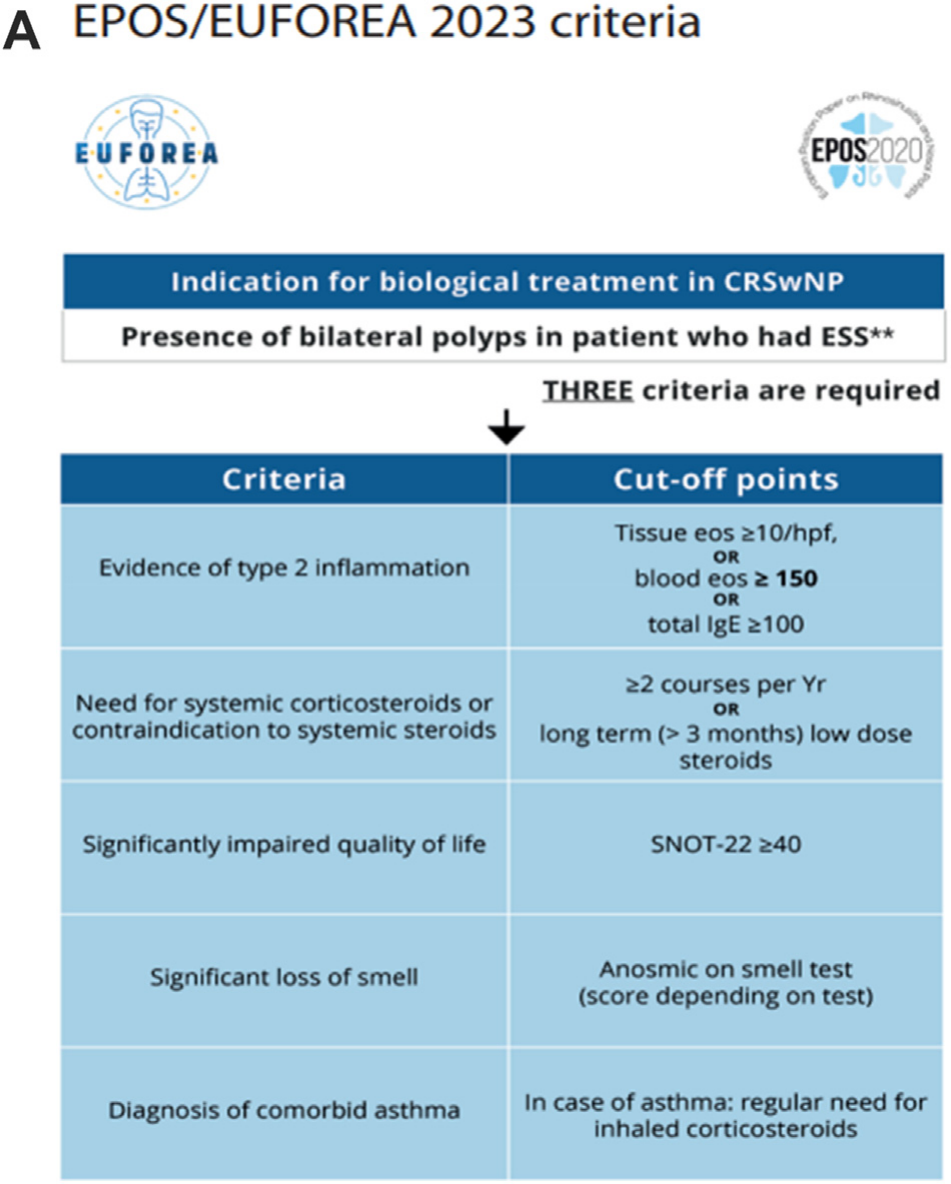

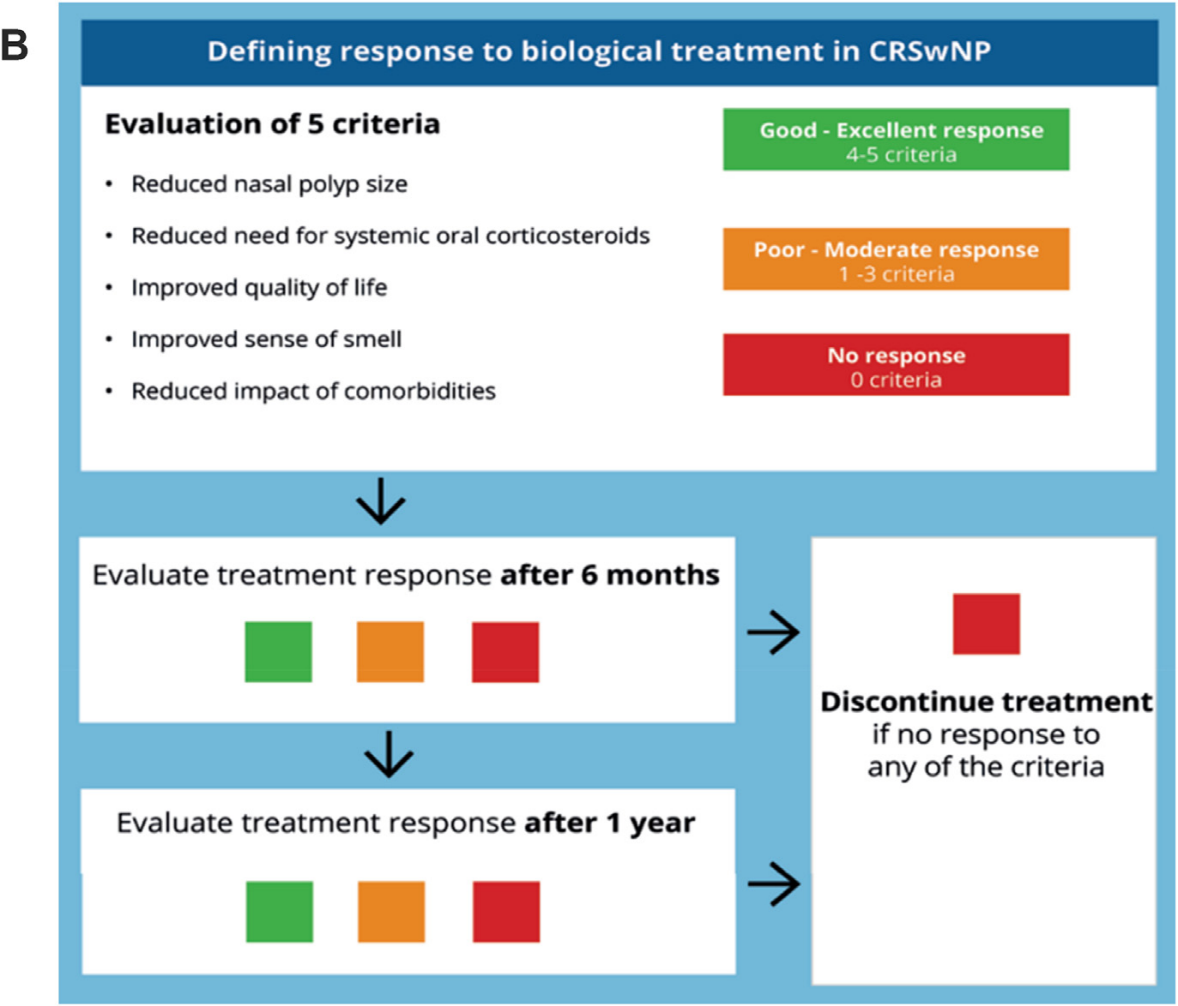
“Actual proposals for prescription (A) and discontinuation (B) of biologicals in CRSwNP” *from Fokkens WJ et al.*^25^

**Fig. 3:**
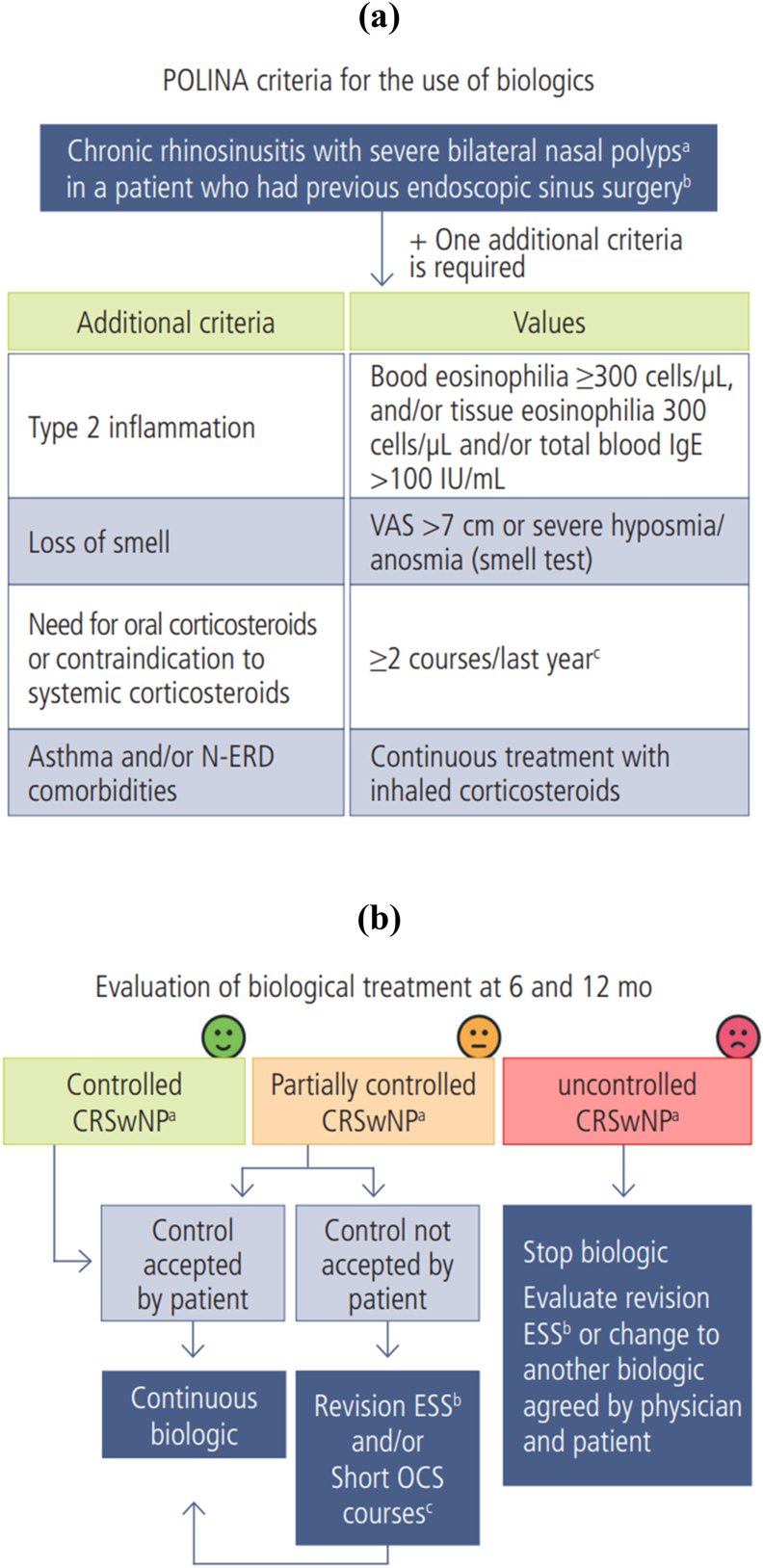
“POLINA Consensus criteria for the use of biologics (A) (Legenda: VAS: visual analog scale; SNOT-22: Sino-Nasal Outcome Test-22; HPF: high-power field; N-ERD: NSAID-exacerbated anti-inflammatory drugs. (a) VAS >7 cm and/or SNOT-22 > 50; (b) Opening of affected paranasal sinuses >6 months; (c) Short courses from 5 days at a dose of 0.5–1 mg/kg/d) and for the evaluation of biological treatment at 6 and 12 months (B) (Legenda: (a) See POLINA control criteria; (b) Endoscopic sinus surgery with opening of affected paranasal sinuses; (c) Oral corticosteroids for 5 days at a dose of 0.5–1 mg/kg/d)”. *from Alobid I et al.*^74^

So far, common sense proposals still remain for the prescription of MAbs in CRSwNP as summarized in Fig. 2 A-B, as suggested previously in the EUFOREA documents.^25^^,^^73^ The recent Spanish Consensus on the Management of Chronic Rhinosinusitis With Nasal Polyps (POLIposis NAsal/POLINA 2.0) is also very interesting and offers many insights and gives important clinical and therapeutic directions.^74^ This consensus aim to translate current knowledge into an easy-to-read practical guide and emphasize those aspects requiring further discussion or with unmet needs owing to the lack of appropriate scientific evidence. An evidence-based systematic review with recommendations was followed using: a standard quality assessment approach (Scottish Intercollegiate Guidelines Network [SIGN] and National Institute for Health and Care Excellence [NICE]), the Appraisal of Guidelines for Research and Evaluation (AGREE II), and Recommendation Excellence (AGREE REX) instruments. The POLINA consensus should therefore be regarded as a document of high quality providing new definitions of control, therapeutic management (including surgery and evaluation of severity), indications for use of biologics, and response. Finally, this guideline focuses on unmet research needs in CRSwNP. Following the international consensus guidelines, the POLINA guideline proposes modified criteria for the indication of biologics in severe CRSwNP and for the assessment of response to biologics at both 6 and 12 months (Fig. 3a and b).

Indeed, the introduction of biologicals as a pharmacological treatment for a disease that has been for long time essentially surgical (or OCS treated), poses many questions, which be solved by both clinical trials and real-world observations:

• Which is the optimal biological for patients with CRSwNP or for patients with CRSwNP and comorbidities (as severe asthma) ?
• Biological first or ESS first?
• How long to use a biological, if it works?
• When to switch from a biological to another?
• How to define a complete remission?
• Who is a super-responder or a poor responder?

Finally, from an economic standpoint, 1 certainly relevant aspect that needs to be carefully evaluated and considered is the cost-effectiveness of therapy with biologic agents compared with other therapeutic approaches, particularly with the surgical approach. As appropriately pointed out by van der Lans et al, to evaluate the position of such expensive treatments for CRS, they should be weighed against the possible gain or savings they would bring.^75^ Indeed, according to its high prevalence, chronicity, frequent exacerbations, and high QoL impact, the socioeconomic cost of CRS is high. In particular, although the CRS direct costs are great, the indirect costs are actually much greater.^76^^,^^77^ Most socioeconomic cost data are from the United States, but very similar results have been founded in Europe and Canada.^78^^,^^79^ Furthermore, patients with CRSwNP demonstrate greater health care resource utilization and costs compared to those with CRSsNP.^80^ Sound data on the cost-effectiveness of biological agents in CRS remain missing. In this context, there is a lack of head-to-head studies comparing different biologics and between biologics and ESS. Theoretically in comorbid patients with CRSwNP and severe asthma, the systemic therapeutic impact of biologics could be greater than EES alone.

Since allergists, ENT specialists, pulmonologists, pediatricians and immunologists participated in the redaction of this article, all of them would search responses to these unsolved questions, by means of a factive cooperation^81^^,^^82^ in linking the different diseases witch may share a same background (severe asthma, CRSwNP, eosinophil esophagitis, eosinophilic pneumonia, atopic dermatitis, and others). The present document is intended to encourage such a similar approach.

## Abbreviations

ARIA, Allergic Rhinitis and its Impact on Asthma; CRSwNP, Chronic Rhinosinusistis with Nasal Polyposis; CRSsNP, Chronic Rhinosinusitis without Nasal Polyposis; ESS, Extended Sinus Surgery; MAbs, Monoclonal Antibodies; OCS, Oral Systemic Corticosteroids; SA, Severe Asthma.

## Author contribution

All the mentioned Authors equally contributed to the design, preparation, writing, and approved the final version. The complete list of the members of ARIA-Italypanel is available at the end of the document.

## Ethics approval

The Authors report that this review is accurate and an objective discussion was performed.

## Disclosure

None by all Authors.

## Confirmation of unpublished work

The authors confirm thet this manuscript is original, it has not been published before, it is not currently being considered for publication elsewhere.

## Funding sources

The authors report no funding sources for the design, prepartion, and writing this article.

## Declaration of competing interest

The authors report no competing interests.
